# Two Recent Autopsies at Cook County Hospital

**Published:** 1887-04

**Authors:** Elbert Wing

**Affiliations:** Pathologist to the Hospital; 3266 Cottage Grove avenue


### HOSPITAL REPORTS.

TWO RECENT AUTOPSIES, AT COOK COUNTY HOSPITAL. REPORTED FOR THE *Journal and Examiner*. *By* ELBERT WING, A.M., M.D., PATHOLOGIST TO THE HOSPITAL.

For the sake of brevity, only those conditions in these cases which are of special interest are reported in the descriptive form in use in the hospital records, the other pathologlcal conditions being mentioned only as they are described in the diagnosis.

Case No. 1.—The patient was brought to the hospital by the police patrol in a state of coma, and died thirty minutes after admission. The officers in charge knew nothing of the history of the case, and no friends appeared to claim the body.

The autopsy was made thirteen hours after death. The body was that of a man about forty years of age, with a moderate amount of emaciation. Rigor mortis was slight, and there was marked icterus in the sclerotics and in the skin about the head, neck and upper part of the thorax. There were no marks of violence, and the abdomen was only moderately distended.

The abdominal cavity contained 750 c.c. of clear, dark brown serous fluid, and each pleural cavity contained 420 c.c. of similar fluid. The appearance and contents of the pericardial cavity were normal.

The right auricle contained a small quantity of dark, clotted blood, and the right ventricle was distended by similar contents. The left auricle and ventricle contained a small quantity of similar blood. The valves of the heart were all slightly thickened. The endocardium was in the condition described as chronic endocarditis, and there were a few small spots of atheroma at the base of the aorta. The muscular tissue of the heart was somewhat pale, and the size of the organ normal.

The visceral and parietal layers of the pleura were united by firm adhesions over almost the entire upper lobe of the right lung, and there were a few firm bands over the rest of the surface of both lungs. There was marked œdema in the upper lobe of the right lung and hypostatic congestion in the lower lobes of both lungs.

In the spleen there were two abscesses, the size of a pea, and the organ was otherwise in normal condition.

On the posterior surface of the left kidney there was a white nodule, which upon section was found half imbedded, and the size of a filbert. This tumor was of a glistening white color, rather soft, contained no large blood vessels, and did not have a hyperæmic border. The cut surface presented an appearance of fine fibrillated, somewhat concentric structure, which, however, could be made to disappear upon stretching.

Upon microscopical examination, this tumor was found to be a round-celled sarcoma. At its circumference there was a moderate degree of acute parenchymatous nephritis, and there was no well-defined line of demarcation between the tumor and the proper structure of the kidney. There were several similar tumors, the size of millet seed, in the vicinity of the large nodule. With the exceptions already mentioned the kidneys were apparently in normal condition.

The liver extended 10 c.m. in the right linea mammalis below the costal cartilages, and was firmly adherent to the diaphragm, duodenum, pancreas and the pyloric end of the stomach. The surface was mottled, with large, bluish-red and icteric patches. The greatest relative increase in the size of the liver was its thickness, which was 15 c.m. About 4 c.m. from the median line of the body, and just below the costal cartilages, there was a marked and somewhat conical swelling of the surface of the liver. Over this area of 4 c.m. there was distinct fluctuation on palpation, and several small, yellowish-white spots, which had the appearance of points in an abscess about to rupture spontaneously. The principal section of the organ was made through this swelling, and revealed an abscess 12 c.m. in diameter in the right lobe, containing thick, stringy, greenish-yellow pus of a slightly sweetish odor. After this cavity was freely washed out, there remained upon its walls a coating of tough, stringy pus, say 10 m.m., thick, from which larger ropes, or strings, of pus hung, which could not be dislodged by a stream of water of considerable force. For a distance of 5 m.m. around the abscess, the liver tissue was markedly hyperæmic. The appearance of the cut surface of the organ generally was that of chronic passive hyperæmia.

The gall bladder was one-third full of bile, and the ductus communis choledochus was freely open.

The stomach and small intestine presented only normal appearances. The mucus and submucous coats of the cæcum and of the lower half of the ascending colon was very extensively ulcerated. The ulcers varied in size, from a pin-head to a lima bean. They were both round and oval in shape, the edges were slightly raised, the bases red, and the flow smooth. In the cæcum they were almost confluent, but they became smaller and less numerous toward the transverse colon. The transverse and descending colon were in normal condition.

With the exception of a considerable œdema of the pia mater, especially marked over the right frontal lobe of the cerebrum, the examination of the brain revealed no pathological conditions.

It is perhaps worth while to call attention to the fact that the abscess of the liver was single, and the ulceration of the colon confined to the upper portion ; while in abscess of the liver, resulting by thrombo-phlebitis from ulceration in dysenter, the abscesses are multiple, and the ulceration more severe at the lower end of the colon.

The sarcoma of the kidney was primary, probably developed, as described by Grawitz, from the internal layer of the capsule of the organ. Such sarcomata very rarely occur.

In consequence of the findings of the autopsy, the following diagnosis was given, viz : Œdema of the pia mater; chronic endocarditis with atheroma ; double chronic pleuritis with double hydrothorax ; œdema of upper lobe of right lung with hypostatic congestion of both lungs ; multiple abscesses of the spleen ; primary sarcoma of left kidney ; abscess of the liver ; colitis with ulceration of cæcum and ascending colon.

Case No. 2 was that of A. B., 35 years old, admitted

November 8th, the diagnosis being typhoid fever. The attack was considered a light one, and the patient was allowed to get up on November 28th. During the month of December he spent his time partly in bed and partly sitting up in a chair. During this period the progress of the case was imperfectly recorded. From January 1st to 12th the temperature ranged from 99° to 100°.2 F. On January 12th, at 12 p. m., the pulse rapidly rose to 120°, the temperature, rectal, to 105°.2, and the respirations to 32. The following record is a copy of the history-sheet of that date, all degrees of temperature given being according to the Fahrenheit scale, and taken in the axilla :

"January 13th, 1:30 a. m., T. 104°.5 ; 2:45 a. m., T. 104°.4 ; 3 a. m. placed in ice pack ; 3:30 a. m. T. 104°.2 ; 4:45 a. m., T. 103°, pack removed ; 4 a. m. T. 103°.5 ; 6 a. m., T. 101°.8; 8 a. m., T. 99°.6; 9:30 a. m., T. 101°.8; abdomen markedly tympanitic and tender, patient vomiting frequently ; 6 p. m., P. 20, T. 101°.4; 9 p. m., P. 120, T. 101°.5 ; 11:45 p. m., T. 98°.6. Jan. 14, 2 a. m., T. 102.8 ; 4 a. m., T. 100°.2 ; 6 a. m., 98°.8 ; in state of collapse all day ; 9 p. m., T. 96°.8, death."

Owing to unavoidable delays, the autopsy was held four days after death. The condition of the organs revealed by the post mortem examination was as follows, viz:

There was chronic myo-endocarditis, with fatty degeneration of the muscular tissue of the heart. Double acute parenchymatous nephritis, with a few firm, white, round spots in the pyramids of the kidney. These spots were as large as a grain of wheat, uniform in appearance throughout their extent, and were what Virchow has called localized interstitial nephritis. Acute parenchymatous hepatitis. Acute parenchymatous splenitis. The abdomen was only slightly tympanitic. There was a small quantity of somewhat turbid serous fluid in the abdominal cavity, which contained a moderate amount of flakes of fibrin. The small intestines were matted together by recent and soft adhesions, and in the left iliac region, near the median line, there was a recent adhesion between the intestine and the abdominal wall. The area of this adhesion was 3 c.m. in diameter. There were several recent, but slight, adhesions betwen the liver and the diaphragm. On the parietal surface of the perito neum there were a number of large, bluish-red patches, the surface of which had a villous or velvety appearance, and in these patches, and also on other parts of the surface, the vessels were filled with blood, and presented a dendritic appearance. That part of the peritoneum covering the intestines presented a similar appearance.

The intestines contained a small quantity of homogeneous, yellow fluid fæces. A small number of the solitary glands, and two of Peyer's patches, were infiltrated and slightly raised above the surface of the intestine. In the ileum, 20 c.m. from the ileo-cecal valve, which point corresponded with that of the adhesion between the intestine and the abdominal wall already mentioned, there was a round perforation of the intestine the size of a slate-pencil. With the exceptions already mentioned, there were no lesions in the intestinal canal.

This case emphasizes very strongly one interesting and one important point in connection with cases of typhoid fever. It is interesting to observe that the number of ulcers in an unmistakable case of typhoid fever may be small—in this case one. It is important to remember that in cases of slight severity, because of the very mild character of the symptoms, physician, nurse and patient are apt to be careless, and thus frequently make them the most dangerous ones.

3266 Cottage Grove avenue.

